# Heart rates, facial expressions and self-reports: a multimodal longitudinal approach of learners' emotions in the foreign language classroom

**DOI:** 10.3389/frai.2025.1604110

**Published:** 2025-10-01

**Authors:** Delphine Guedat-Bittighoffer, Abderrazzaq Moufidi, Jean-Marc Dewaele, David Rousseau, Hugo Voyneau, Pejman Rasti

**Affiliations:** ^1^UFR Lettres Langues et Sciences Humaines, Université d'Angers, Angers, France; ^2^Laboratoire Angevin de Recherche en Ingénierie des Systémes (LARIS), UMR INRAe-IRHS, Université d'Angers, Angers, France; ^3^Institute of Education, University College London, Birkbeck, University of London, London, United Kingdom

**Keywords:** heart rates, facial expressions, foreign language enjoyment, foreign language classroom, emotional contagion, self-report

## Abstract

Emotions in educational settings are often studied through self-reports or lab experiments, limiting insights into their real-world dynamics. This study examines learner emotions in authentic foreign language classrooms using a multimodal longitudinal approach. Over 16 consecutive sessions, we collected heart rate (HR) signals, emotional facial expressions (EFE), classroom observations, and self-reports on enjoyment, anxiety, and boredom to capture both physiological and self-perceived emotional responses. Rather than aggregating data across students, we focused on individualized emotional patterns to understand variations in emotional experiences. Each dataset included extensive video recordings, continuous HR monitoring, detailed observational notes, and post-session questionnaires, providing a high-resolution picture of emotional dynamics. Using unsupervised clustering techniques, we identified key emotional episodes—peaks and drops in physiological arousal (heart rate variation) and facial expression—relative to individual emotional baselines. These moments were cross-referenced with classroom observations and self-reports for validation. Findings highlight moments of positive emotional contagion during peer interactions, emphasizing the social dimension of language learning. This multimodal approach captures the interplay of physiological, behavioral, and subjective responses, offering a scalable method for studying classroom emotions. Methodologically, it demonstrates how multimodal analytics can uncover transient emotional states in real-world settings, while practically informing adaptive teaching strategies, such as leveraging peer interactions to enhance engagement or reduce anxiety. By integrating physiological, behavioral, and subjective data, this study provides a comprehensive framework for understanding the affective dimensions of learning.

## 1 Introduction

The need for interdisciplinary research has never been greater in the field of foreign language education and applied linguistics (Yu, 2022). While researchers broadly agree that interdisciplinary perspectives allow the creation of “new knowledge frameworks” (Sauvage and Nourrit, 2022), there is no denying that “Even today, it is not so simple to transcend these disciplinary boundaries to build an interdisciplinary, collaborative, and relevant scientific approach” (Sauvage and Nourrit, 2022). While a fair amount of interdisciplinary research has already been carried out in applied linguistics and psychology, there still is much to be done. For example, most research on learners' emotions in Foreign Language (FL) classes is based on self-reported data collected from learners after class through interviews, questionnaires and diaries. While there is nothing wrong with self-reports (Dewaele, 2023), some researchers have argued out that these approaches might be insufficient to fully capture the dynamic and fluctuating nature of emotions in the classroom (Gregersen et al., 2014). MacIntyre (Mackey and Gass, 2023) suggested also that the field needs a new type of study based on multimodal data collection, combining physiological data with ratings of emotions while performing a task, and answering questions about the spikes and dips in a subsequent interview.

Recent advances in technology have made it possible to collect and analyze multiple data streams in real-time, enabling a more sophisticated triangulation of learners' dynamic emotions, experiences of flow, neural activities and facial expressions (Hoemann et al., 2023; Nozawa et al., 2021; Tonguç and Ozkara, 2020). While there is nothing wrong with self-reports, there is an inherent limitation because not everybody is equally capable of verbalizing what they feel (Barrett, 2017). Individuals with low levels of emotional intelligence may give very broad indications on the valence of their emotions (good/bad) while those with higher levels of emotional intelligence detect their own emotions in more detail and nuance, and therefore provide a much more accurate picture of their various interacting emotions. One way to mitigate this source of variation, which could lower the quality of the self-reported data, is to complement it with various physiological measures. We fully agree with the view that multimodal approaches “will make it possible to model emotion in higher dimensionality, and answer fundamental questions about how biological, mental, and contextual features are related over time” (Hoemann et al., 2023).

The present study adopts a cutting-edge multimodal approach, combining Heart Rate (HR) monitoring, Emotional Facial Expressions (EFE) analysis, classroom observations, and self-report questionnaires to assess students' enjoyment, anxiety, and boredom over several weeks. We argue that the use of sophisticated data analysis techniques and advanced statistical methods can lead to a better understanding of the dynamics of learner emotions while they perform tasks together in the classroom. This multi-pronged approach could ultimately lead to the development of a robust tool that can provide real-time feedback on student emotions.

The remainder of this paper is organized as follows. Section 2 reviews related work on multimodal approaches to studying learner emotions in foreign language classrooms, with a focus on the dimensions of enjoyment, boredom, and anxiety. Section 3 details the study design, participants, instruments, and data analysis pipeline. Section 4 presents the experimental results, including convergences between modalities and individual case analyses. Section 5 discusses the findings in relation to previous research and the broader implications for language teaching and learning. Finally, Section 6 concludes the paper and outlines potential directions for future work.

## 2 Related works

The theoretical basis of the present study is inspired from Gobin et al. (2021). The authors argue that an emotion arises in response to a particular emotional situation (Lazarus, 1991) that is more or less significant. Individual responses to this event consist of three components: (1) a component of physiological arousal (motivational dimension) that refers to physiological responses, meaning all the body's internal reactions; (2) a component that corresponds to motor expression and refers to the visible verbal or non-verbal manifestations of emotion, with the most common expression being “Emotional Facial Expressions” (EFE); (3) a final component called subjective feeling that reflects subjective awareness and englobes the cognitive-experiential responses to emotion. It consists of all cognitive processes related to the perception of the emotional situation, which the individual can verbalize and explain.

### 2.1 Multidimensionality of emotions in FL courses

Research into the use of multimodal data for analyzing learner's emotions, particularly in educational contexts, has evolved significantly since the 2000s. The idea of integrating physiological and behavioral data to understand dynamic interactions between motivation, emotions, and Willingness to Communicate (WTC) across educational environments is not new (De Bot et al., 2007) but technological developments have allowed researchers to use sophisticated tools that are increasingly affordable to applied linguists. Here, we review some key studies.

D'Mello et al. (2012) explored the use of multimodal data to monitor engagement and learning in real-time. This research utilized a combination of EFE, body posture, and interaction logs to understand how students engage during learning activities in various environments, including classrooms and online platforms. The researchers used advanced and expensive equipment which would be unaffordable to most researchers.

Another study, conducted by Tonguç and Ozkara (2020), employed a cheaper facial recognition tool with 67 students during basic information technology courses, using a camera placed in each student's computer. The materials used during the lecture were reflected on students' computer screens. EFE were analyzed and digitized to identify seven emotions/feelings: disgust, sadness, happiness, fear, contempt, anger, and surprise based on Ekman's theory of universal emotions (Ekman, 1992). This approach has been strongly criticized for being too static and essentialist. Barrett (2017) and Gendron et al. (2018) have proposed an alternative approach, the theory of constructed emotions which posits that emotions are shaped by the broader cultural context and the more specific social context in which the emotion unfolds. They also reject the idea that emotions have universal unique “fingerprints,” in other words, a smile does not automatically reflect happiness and a scowl does not always signify anger. The dynamic theory of constructed emotions is particularly appropriate when studying multilingual and multicultural individuals.

Adopting a dynamic approach, Gregersen et al. (2014) conducted a multimodal study on three high-anxiety and three low-anxiety learners of Spanish FL [based on scores on Horwitz et al. (1986) FL Classroom Anxiety Scale]. The authors combined physiological data (heart rates), idiodynamic data (anxiety ratings), and interviews about the fluctuations. Participants did a 3-minute oral presentation in Spanish. They wore heart monitors and, immediately after the task, they provided 42 anxiety ratings times on a scale from +5 to –5 while viewing their presentation on a computer. They then explained to the researchers why the spikes and dips in anxiety had occurred. Increased heart rates were positively correlated with anxiety ratings. The high anxiety participants reported difficulties in vocabulary retrieval as the main cause for their anxiety. Low anxiety participants used strategies to mitigate this. They had practiced the presentation in the preparation stage rather than attempting to memorize it. The study used various sources of data to highlight the dynamic nature of a single emotion but ignored the fact that participants may have experienced other emotions, which could also have affected heart rates.

The pioneering neurological study by Nozawa et al. (2021) was the first to peer into learners “black box,” namely their brain waves, as they were performing tasks in class. The researchers focused on two intact English FL classes in Japan with two groups of four learners each (totaling 16 students). They adopted a multimodal approach, examining the interbrain synchronization among learners working in pairs and the similarity of the flow state dynamics during collaborative learning. Prefrontal neural activities were measured using a wireless functional near-infrared spectroscopy device placed on the students' heads. Additionally, the researchers asked the learners to watch recorded videos of the classes and to evaluate their own flow levels on a scale from 1 to 7 every two minutes. The study required advanced technology and know-how to process the neurological data in a single cortical area. The authors admit that the correlation between self-reports and interbrain synchronization does not imply causation as there may be “hidden variables.” Dewaele (2023) explained there are different recent studies which try to: “catch the elusive emotional phenomena that swirl around task performance.” (p. xviii). Lambert et al. (2023) propose to resort to electroencephalography (EEG), eye tracking (ET), electrodermal activity (EDA), and automated facial expression analysis (FEA). MacIntyre (2023) revisits and develops his idiodynamic method, which allows for detailed and accurate observation of the emotional fluctuations experienced by learners while performing tasks in language classes.

These above studies highlight the diversity of methodologies and technologies used in the multimodal analysis of learners' emotions in the FL classroom. All relied on supervised learning approaches that require extensive annotation and labeled data, which can be resource-intensive, time-consuming and expensive. In contrast, our approach employs unsupervised Gaussian Mixture Model (GMM) clustering, which identifies specific moments without requiring pre-labeled data. This allows us to account for individual differences in learners' physiological and emotional patterns while reducing the need for extensive manual labeling, making the methodology more efficient and adaptable.

### 2.2 Anxiety, boredom, and enjoyment in FL classes

The introduction of positive psychology in the field of FL acquisition with the publication of MacIntyre and Gregersen (2012) made researchers aware that there had been a long-term exclusive focus on negative emotions in FL classrooms (Petiot and Visioli, 2022) and anxiety in particular. Its popularity among researchers had been boosted by Horwitz et al. (1986) whose 33-item Foreign Language Classroom Anxiety Scale (FLCAS) covered physical symptoms of anxiety, nervousness and lack of confidence in the FL class. They defined FLCA as “a distinct complex of self-perceptions, beliefs, feelings, and behaviors related to classroom language learning, arising from a uniqueness in the language learning process.” The main cause of FLCA is the inability to project an accurate image of themselves in the FL and the fear of coming across as clumsy and inauthenthic (Horwitz, 2017). FLCA grows gradually through repeated anxious experiences in the FL classroom. As such, FLCA starts as being a situation-specific state and it gradually becomes more stable and trait-like (Horwitz, 2017).

A meta-analysis by Botes et al. (2020) of 67 studies based on the FLCAS has shown that FLCA is moderately negatively linked to FL performance and progress. High FLCA was linked to lower general academic achievement, speaking, listening, reading and writing performance in the FL. Students suffering from high FLCA have lower degree of Willingness to communicate (WTC) and may even prefer to hide and remain silent in the classroom (King, 2013). This withdrawal from classroom interactions slows their progress in the FL.

Another negative emotion, frequently present in FL classrooms, is boredom (Pawlak et al., 2024). Li et al. (2023) described boredom as being characterized “by negative valence, low arousal and being achievement-related activity-focused.” It emerges when a classroom activity or task is perceived as irrelevant and when learners feel helpless and fatigued because the activity is either too easy or too difficult (Agrawal et al., 2022; Li et al., 2023). Bored learners lose their confidence and suffer from a perceived lack of control. This lowers their WTC, and undermines both their short-term and longer-term motivation and well as their overall engagement in the FL activities. Learners' boredom can also originate in the teacher's inability to hide their own boredom (Pawlak et al., 2024). Li et al. (2023) developed 32-item Foreign Language Learning Boredom (FLLB) scale consisting of 7 factors. The first factor was named Foreign Language Classroom Boredom (FLCB) and consisted of 8 items. FLCB has been used independently in later research. Dewaele and Li (2021) showed that teacher enthusiasm can counter learners' FLLB, increase their enjoyment and stimulate their engagement. Li et al. (2022) found enjoyment and boredom to be strongly negatively correlated. Unsurprisingly, a negative relationship exists between FLLB and FL achievement (Li and Dewaele, 2024).

Researchers increasingly agree that positive emotions such as Foreign Language Enjoyment (FLE) should be part of a more holistic picture and they reject the deficit view of FL learners Dewaele and MacIntyre, (2014). The authors referred to Csikszentmihalyi (1990) who noted that enjoyment (and sometimes flow) emerges when a person manages to complete a challenging task, reach a state of full concentration, perform a task with clear goals, and receiving immediate feedback on the performance. (Dewaele and MacIntyre 2014) developed the 21-item FLE scale, which was followed by a shorter 9-item psychometrically validated scale (Botes et al., 2021). While a majority of the longitudinal studies on FL emotions focused on change over a period of weeks and months, a smaller number of studies have focused on fluctuations over shorter time spans. Boudreau et al. (2018), for example, used the idiodynamic method to investigate second per second fluctuations in FLE and FLCA. Anglo-Canadian participants completed a one-minute speaking task in French FL and then watched the recording and reported their levels of both emotions for every second. Values were found to vary considerably during that minute and were later commented on by participants who pointed to linguistic difficulties or to fleeting moments of anxiety, enjoyment or boredom. Elahi Shirvan and Talebzadeh (2018) also used the idiodynamic approach to investigate the fluctuations in FLE of 7 Iranian EFL university learners participating in conversations on simple and more difficult topics. The results showed strong intra- and inter-individual variation linked to the conversational topics. Adopting a multi-case study design, Elahi Shirvan et al. (2020) investigated fluctuations in FLE over different time spans, ranging from seconds with the idiodynamic method, to minutes, weeks and months. The researchers used low tech “Enjoymeters” (pieces of paper with thermometer-shaped figures ranging from 0 to 10 indicating the level of FLE) to capture variation in FLE for periods of 5 minutes. Participants were two Iranian EFL students in the classroom. FLE among these two students was found to fluctuate differently over different timespans. The variation was found to be linked to unique social and personal factors, such as the ability to be creative, the appropriate challenge, the opportunity for authentic communication in English with peers, the teacher's ability to be supportive, humorous and establishing a positive classroom climate. Being laughed at by peers for making a mistake could cause a sudden drop in FLE and a spike in anxiety. Bielak and Mystkowska-Wiertelak (2024) also used the idiodynamic methodology to investigate fluctuations in FLCA and FLE in 10 Polish EFL learners working in pairs and group. Their interactions were video recorded and then viewed and rated second per second for FLE and FLCA. In later stimulated-recall interviews, they discussed the causes of the fluctuations and the emotion regulation strategies they deployed to control them. The two emotions showed brief periods of stability followed by highly idiosyncratic levels of fluctuation. Levels of FLCA were found to fluctuate more than FLE but the triggers for the fluctuation in both emotions overlapped substantially. Causes for FLCA included the awareness of having made specific errors, frustration at the lack of linguistic sophistication, and ignoring task instructions. FLE was found to be linked to the quality of the peer's performance and a productive collaboration. Taking the floor and deploying new knowledge caused peaks in both FLE and FLCA while yielding the floor cause a dip in both emotions.

Dewaele and Pavelescu (2019) used a multiple case study approach to investigate the relationship between FLE, FLCA, and WTC in two Romanian secondary school EFL learners. Qualitative data included classroom observations and semi-structured interviews on the emotional sources of fluctuation in participants' WTC in the English classroom. FLE and FLCA were found be influenced by a range of contextual factors including seating arrangements, course material and conversation topic which shaped their WTC in dynamic and unique ways.

The meta-analysis by Dewaele and MacIntyre (2022) showed that FLE is strongly positively correlated with WTC. Moderate positive relationships emerged between FLE and FL academic performance. The crucial awareness that emerged from previous research is that learner emotions do not exist in isolation. Studies on large samples reveal positive correlations between positive emotions, and between negative emotions, combined with negative correlations between positive and negative emotions. This suggests that there is a strong probability that students who are enjoying themselves are less likely to suffer from anxiety and boredom. As the idiodynamic studies show, the emotions are constantly connected with each other, with motivation and all are linked to the immediate classroom environment and the wider social context (Dewaele and MacIntyre, 2022; Li and Dewaele, 2024; Waninge et al., 2014). The teacher is central in this context and his/her perceived enthusiasm or happiness can cause a wave of positive emotional contagion (Li and Dewaele, 2021; Moskowitz and Dewaele, 2021; Talebzadeh et al., 2020). A teacher who cannot hide his/her boredom will spread this negative emotion to students (Pawlak et al., 2024). The relationship with peers and with a specific partner in pair-work will also shape individual learner emotions. Collaborating with someone who is very anxious or bored will drag down the enthusiasm of learners who actually enjoy the activity. On the other hand, working with an empathic, friendly, funny partner might boost learners' positive emotions and lower their negative emotions. The task and activity itself will also shape learners' emotions, as they may -or may not- enjoy it and grow bored with it if it lasts for too long (Li and Dewaele, 2024).

To sum up, this short overview of the existing literature shows a field in rapid transition because of the emergence of a holistic understanding of learner emotions and of their dynamic nature, combined with technological innovations. Studies using the idiodynamic method used material collected laboriously over a period of no more than a few minutes in a lab. They also focused on no more than two emotions in order not to overwhelm participants. Very few of these studies included a physiological measure. We thus argue that there is an urgent need for multimodal studies focusing on a larger number of emotions tracked for longer periods in real-world FL classroom environments using economical and efficient techniques. Adopting a non-supervised approach relying on non-intrusive, low-cost instruments with robust data processing techniques could lead to the development of a scalable and replicable system.

The current study thus proposes to analyze emotions of a small number of participants over a period of several weeks. We adopted a design similar to that of Nozawa et al. (2021) with external technical or digital measurement instruments (pulse oximeter and camera placed in the classroom) and self-perception data collected from students (enjoyment, boredom, and anxiety questionnaires).

## 3 Method

### 3.1 Research questions

We will focus on the following research questions:

**RQ 1:** Do multimodal methods of data collection, i.e. measuring HR, EFEs, and class observations allow researchers to gain an overall view of the emotions experienced by students in language classes?
**RQ 2:** Do the scores obtained through the FLE, FLCB, and FLCA scales, along with the learners' responses to the open-ended questions, help to better understand and interpret their physiological measures (HR variation) and their EFE linked to the class observations?

### 3.2 Study design and participants

The project took place at the French language center for foreigners at the University of Angers. Participants were preparing for the University Diploma in French Studies (DUEF) at the beginner level, A2 according to the Common European Framework of Reference for Languages (CEFR). The courses were held over four days in the second semester of the academic year, from February to April 2023. The study was conducted over 16 sessions of 2 hours and 40 minutes each (2 × 1 h and 20 min). Of the eleven students in the class, seven provided consent; however, only those consistently present across the majority of sessions—defined here as attending at least 50% of the 16 total sessions—were included in the analysis (three students: pseudonyms “Mitch,” a 21-year-old American, attended 14 sessions out of 16 sessions; “Zeynep,” a 23-year-old Turkish, attended 14 sessions out of 16 sessions; and “Oksana,” a 23-year-old Ukrainian, attended 13 sessions out of 16 sessions). This choice reflects our methodological emphasis on individualized emotional patterns rather than aggregated group data. By concentrating on a small number of consistently present participants, we were able to collect extensive multimodal data—continuous HR monitoring, facial expressions, video recordings, detailed classroom observations, and post-session questionnaires—allowing for a high-resolution, longitudinal analysis of emotional dynamics over time. A research team member took comprehensive notes for all 16 sessions. During each session, both their HR signals and EFE data were collected, and at the end of each session participants completed a questionnaire. For all attended sessions, the three modalities—HR, EFE, and self-reports (SR)—were obtained; no sessions contained partial modality data.

### 3.3 Ethical considerations

This study was conducted in accordance with the Declaration of Helsinki and institutional ethical standards. All participants voluntarily participated and provided signed informed consent before data collection and analysis. They explicitly agreed to the recording of their sessions, the measurement of their heart rate, and responding to a survey at the end of each session for research purposes. All procedures were designed to respect participants' rights, privacy, and well-being. No identifying information was collected or published.

### 3.4 Instruments

Three types of instruments were used to tap into learners' physiology, EFE and self-report of FLE, FLCB, and FLCA.

#### 3.4.1 Physiological reaction: measurement of HR variation

**Figure 1:**
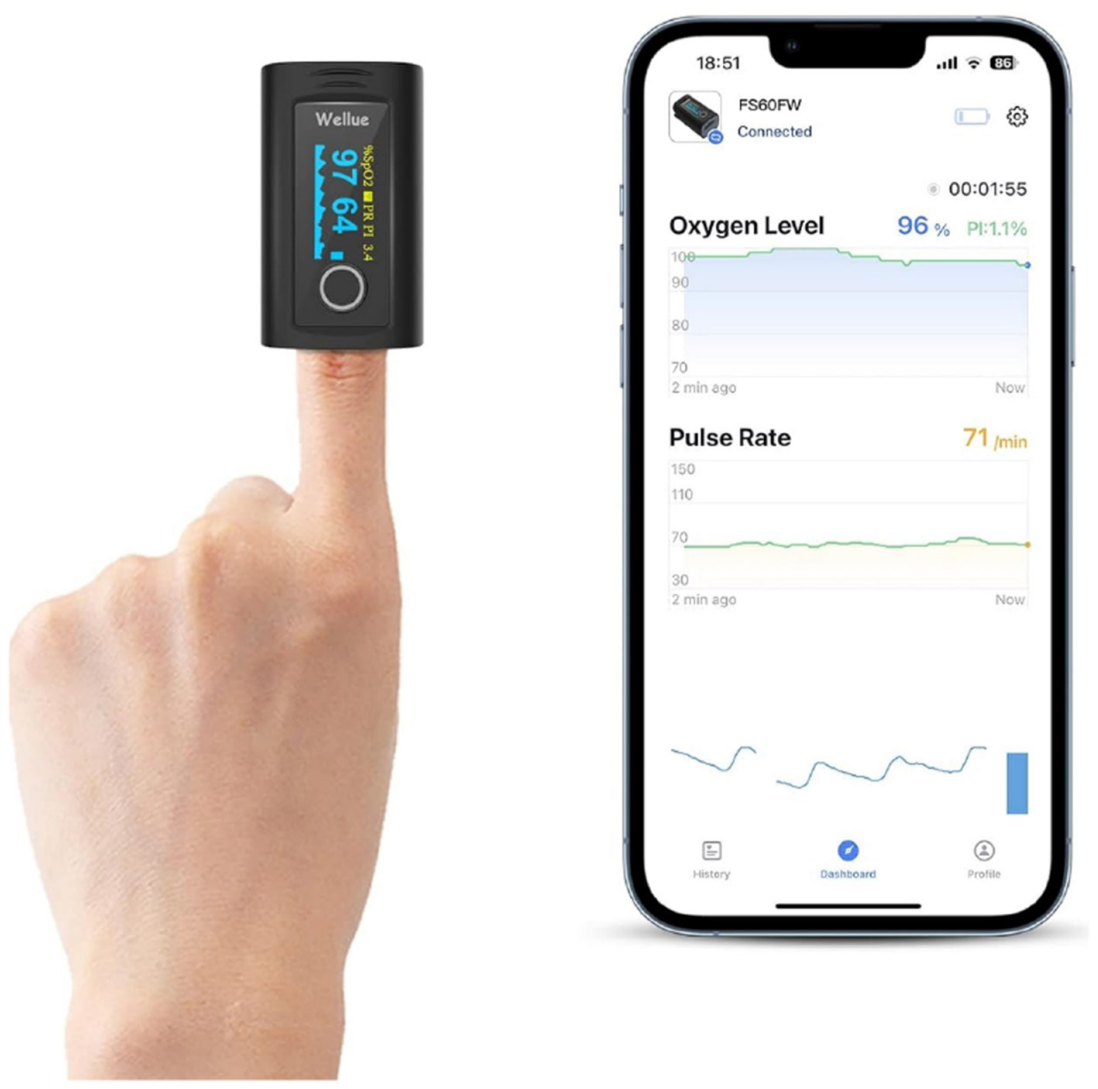
Left: Oximeter used to record the heart rate beats of the students. Right: ViHealth application that receive the data from the oximeter.

An electrocardiogram (ECG) measures ECG signals, which can be used to predict numerous features such as heart rate (HR), interbeat interval, and HR variability (Kim and André, 2008). For our task, we selected an affordable wearable device called the “Fingertip Pulse Oximeter,” with characteristics detailed in Figure 1. A pulse oximeter measures heart rate by detecting pulsatil changes in blood volume using the photoplethysmogram (PPG) signal. The device emits red and infrared light through the finger, and a photodetector on the opposite side measures the transmitted light. The pulsatile component of blood flow generates a PPG waveform, representing variations in blood volume with each heartbeat. The heart rate is calculated by measuring the time interval between successive peaks in the PPG waveform, determining the number of heart beats per minute (Wukitsch et al., 1988). This method is reliable and accurate for HR up to 155 bpm, suitable for non-strenuous activities (Wukitsch et al., 1988; Iyriboz et al., 1991). Therefore, this instrument meets our needs as it is cost-effective and user-friendly. Students can pair the oximeter with the ViHealth application via Bluetooth, and the recordings are stored on the student's phone in PDF format.

#### 3.4.2 Expressive behavioral responses: EFE

To collect visual data for recognizing student EFE, we selected an affordable camera, the Razer Kiyo Pro (C100). At the start of each session, the camera was positioned to capture the whole classroom, with all participants visible in the frame. Video was recorded in RGB at 25 fps (1,280 × 720 pixels). Individual faces were detected and cropped from the classroom video using a FaceNet-based detection method (Schroff et al., 2015), enabling separate emotion analysis for each participant. Frames of insufficient quality or containing occlusions were excluded from the analysis.

#### 3.4.3 Cognitive-experiential responses: the enjoyment, boredom, and anxiety questionnaires

At the end of each of the 16 sessions, we administered the short version of the enjoyment questionnaire (Botes et al., 2021) to the three volunteers retained for the study. This questionnaire consists of nine items that assess enjoyment in the French FL class across three dimensions: teacher enjoyment (e.g., “The French teacher is kind”), personal enjoyment (e.g., “I am proud of my progress in French”), and social enjoyment (e.g., “We support each other in the French class”). Participants also filled out the 8-item sub-dimension Foreign Language Classroom Boredom (FLCB) (Li et al., 2023). These items address lack of concentration, fatigue, and restlessness, such as “My mind begins to wander in the French class.” Finally, participants filled out the 8-item short form of Foreign Language Classroom Anxiety scale (S-FLCAS), employed by Dewaele and MacIntyre (2014) and validated by Botes et al. (2022a,b). These items refer to physical symptoms of anxiety, nervousness, and lack of self-confidence. Two items refer to low anxiety, such as “I am not afraid of making mistakes in the French class,” and six items indicate high anxiety, such as “I become nervous and confused when I speak in my French class.” Items were accompanied by a 5-point Likert scale (1. strongly disagree, 2. disagree, 3. neither agree nor disagree, 4. agree, 5. strongly agree). The closed items were complemented by two open-ended questions allowing students to freely express their feelings and emotions in their own words. These two questions were concrete, asking students to describe a specific situation in class where they felt really good, a moment when they felt bad, and what they precisely felt at that moment. The qualitative material gathered in this way allowed us “to hear the voices of participants, free from the shackles of the Likert scale items” (Dewaele, 2023). Dörnyei (2007) encouraged researchers to include open questions in questionnaires because they “can provide a far greater richness than fully quantitative data.” The two open-ended questions could be answered in French or English. Thus, Oksana and Mitch responded in English, while Zeynep responded in French. The quantitative part of the questionnaire was used for purely illustrative purposes as no inferential statistics could be calculated. Combined with the answers to the open-ended questions, they forced participants to think about the FLE, FLCA, and FLCB in the classroom and provided a basis for the interviews.

All study materials, including the complete set of questionnaire items are available from the authors upon request.

### 3.5 Data analysis

#### 3.5.1 Proposed pipeline for the HR and the EFE

**Figure 2:**
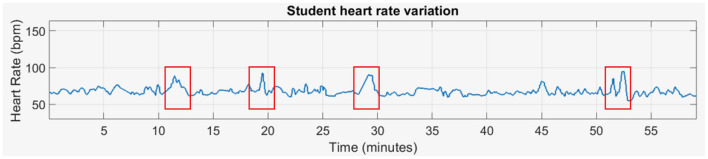
Heart rate variation showing specific deviations from typical patterns.

To monitor student engagement, we combined physiological signals, specifically heart rate (HR) variations, with behavioral signals, such as emotional facial expressions (EFE). While previous studies (Wang et al., 2022; Shu et al., 2018; Poria et al., 2017; Wu, 2023) have highlighted HR data as a reliable measure of physiological states, relying solely on one modality may overlook important behavioral cues that provide additional insight into engagement. EFE can capture cognitive and emotional states that may not be fully reflected in HR data, offering complementary information. By integrating both HR and EFE, we can leverage the strengths of each modality, as they together offer a more holistic view of student engagement. To effectively combine these two sources of information, we adopted a decision-level fusion approach, where HR and EFE are processed independently to detect anomalies. The final decision on engagement incorporates both modalities when available, but the method remains robust even in the absence of one modality. This strategy ensures flexibility and improves the accuracy of engagement detection by compensating for limitations in any single data source, offering a more comprehensive and reliable system. The pipeline consists of two separate sub-pipelines, each dedicated to processing a different modality independently. Given the temporal nature of HR modality, the preprocessed HR data from each session is segmented into equal frames of duration T with a 50% overlap. These segments are then processed by a hand-crafted feature extractor to reduce the high dimensionality of the signals. For each student, the feature vectors from all sessions are clustered in an unsupervised manner into two groups: normal moments, representing most of the time, and Specific Moments (SM), indicating deviations from the norm. We called these SM because they were identified as instances that deviated from the typical patterns observed for each student. These patterns, derived from their data across sessions, served as reference points to highlight unusual physiological or behavioral responses. Figure 2 illustrates these typical patterns and how they were used as baselines to detect significant deviations.

We therefore wanted to understand precisely what was happening for them at those specific moments by correlating these SMs with classroom observations and triangulating with the questionnaires. This approach of combining HR variation analysis and EFE detection introduces a personalized methodology that accounts for individual variations in health status and cultural background. Since resting heart rate (HR) can be influenced by various factors, this approach provides a more accurate and context-sensitive assessment of each student's physiological data (Shu et al., 2020). Simultaneously, the video output from our RGB camera, initially recorded at 25 frames per second (fps), was downsampled to 1 fps and preprocessed to track only the individual corresponding to the HR measurements. The decision to downsample to 1 fps was driven by the need to balance computational efficiency and the temporal resolution of EFE. Since significant changes in facial expressions—defined as noticeable deviations from the learner's most frequent or typical expression during the session—typically occur over a span of seconds rather than milliseconds (Shu et al., 2018), capturing frames at 1 fps is sufficient to detect these changes without unnecessary redundancy.

This approach reduces the computational load while still providing the necessary granularity to accurately track and analyze facial expressions in sync with the HR data. Each face image is then fed into a pre-trained network designed to recognize seven emotions (angry, disgust, fear, happy, sad, surprise, and neutral). The resulting feature vectors are concatenated across all sessions and clustered similarly to the HR signal data to identify SM based on facial expressions. The identification of these seven emotions primarily allowed us to detect deviations from the learner's most frequent emotional facial expression observed during the sessions, rather than categorizing fixed emotional states. This approach aligns with Feldman Barrett's theory (Barrett, 2017) of constructed emotions, which suggests that categorizing emotions into fixed labels is less meaningful than identifying variations from an individual's baseline behavior. What matters is identifying the most frequent EFE for the learner and seeing when there is a variation, called also SM, compared to this standard emotion for them.

**Figure 3:**
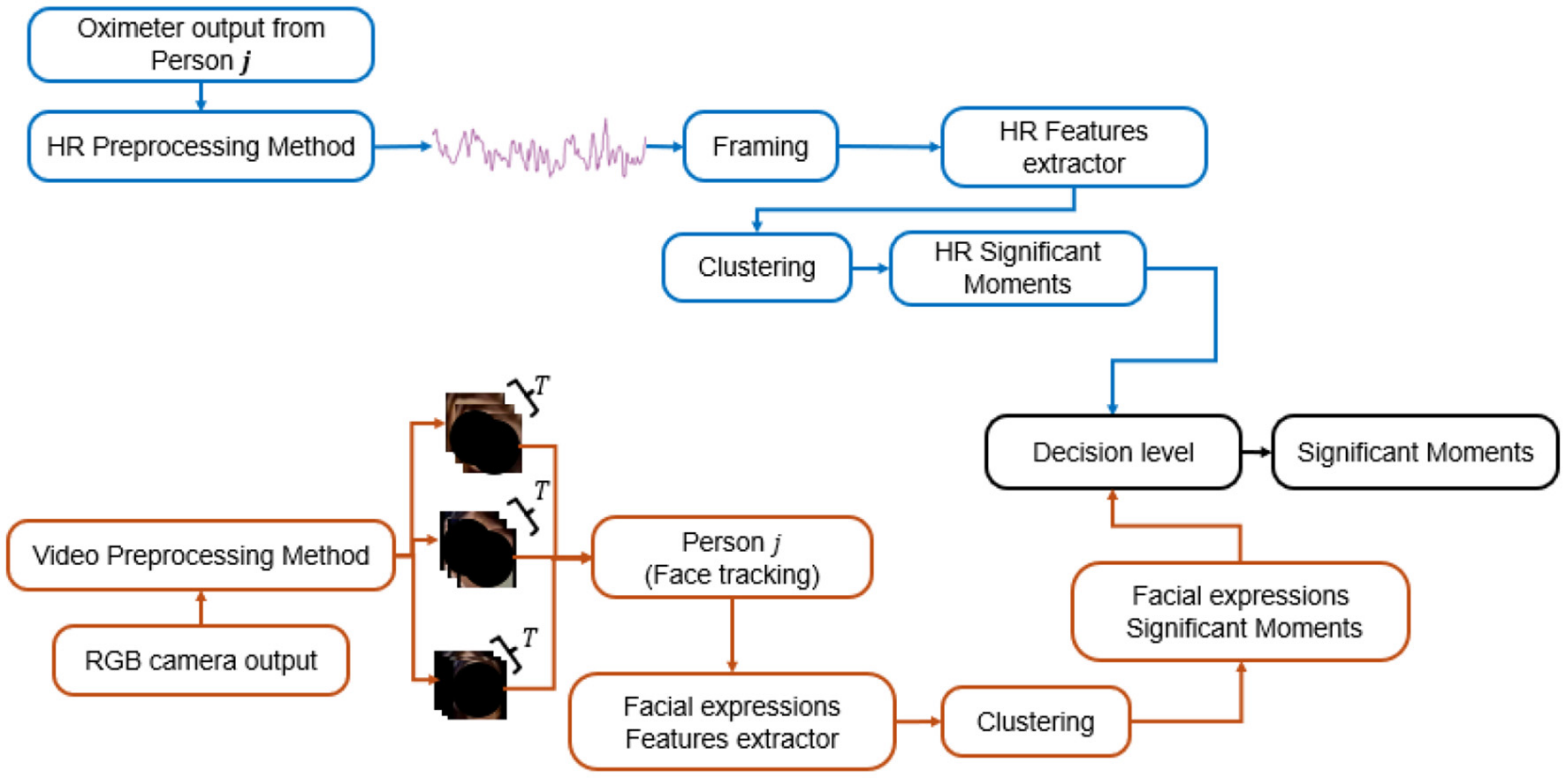
Our proposed pipeline method to detect SM during a teaching session.

Our complete processing pipeline for these two modalities is shown in Figure 3. At the decision level, outcomes are determined by combining information from both modalities whenever available, with greater weight assigned to HR because physiological signals such as HR, regulated by the autonomic nervous system, are generally more objective and less susceptible to voluntary control. In contrast, facial expressions can be consciously suppressed or influenced by factors such as head pose, lighting, or occlusion (Shu et al., 2020). If only one modality is available, the decision is based solely on that source; when HR is present, it takes precedence over EFE.

Having presented an overview of the pipeline, including the independent processing of HR and EFE data and their fusion at the decision level, we now focus on the technical details of the experimental setup. This includes the temporal segmentation of HR signals, the feature extraction process, and the steps involved in clustering and detecting SMs for both modalities.

#### 3.5.2 Experimental setup for the HR and EFE

In the following paragraph, we introduce the experimental setups on subject-dependent to monitor students' emotions using two modalities: HR signals and facial expressions. For the HR signal, due to its temporal nature, we should establish a specific time window T on which we can apply the HR statistical features ensuring a T value that does not affect the emotional decision. According to the literature (Wu, 2023; Kreibig, 2010) time interval between an emotional stimulus and the subsequent physiological response varies due to factors such as individual differences and signaling modality. This variability complicates the task of defining a suitable window size for emotion recognition systems. Kreibig found that the most common average time intervals for physiological responses were 60 s and 30 s in a survey of 134 publications (Wu, 2023; Kreibig, 2010). Other common average intervals were 0.5*s*, 10*s*, 120*s*, 180*s*, and 300*s* (Wu, 2023). Therefore, for the HR signal processing, we choose to work on window of duration *T* = 60*s*, 90*s*, 120*s*, 150*s*, 180*s*, and 210*s* with a 50% overlapping (a hyperparameter that can variate from a physiological signal to another Wu, 2023). The heart rate signal for each student in each session was segmented into windows of length T with a 50% overlap between consecutive segments. This overlap was chosen to enhance the detection of transient physiological changes, especially those occurring near window boundaries, and to ensure temporal continuity in the data. Such an approach has been shown to be effective in emotion recognition from physiological signals, as it preserves short-term fluctuations that are important for accurate classification (Yu et al., 2023).

From each window, we extracted a 6-dimensional feature vector summarizing HR dynamics: mean (μ) and standard deviation (σ); mean absolute speed (μ_*v*_) computed from the first-order finite differences of the HR sequence; normalized absolute speed (μ_*v*_/σ); mean absolute acceleration (μ_*a*_) computed from second-order finite differences; and normalized absolute acceleration (μ_*a*_/σ) (Shu et al., 2020). These features convert the raw time series into a multidimensional representation suitable for clustering.

**Figure 4:**
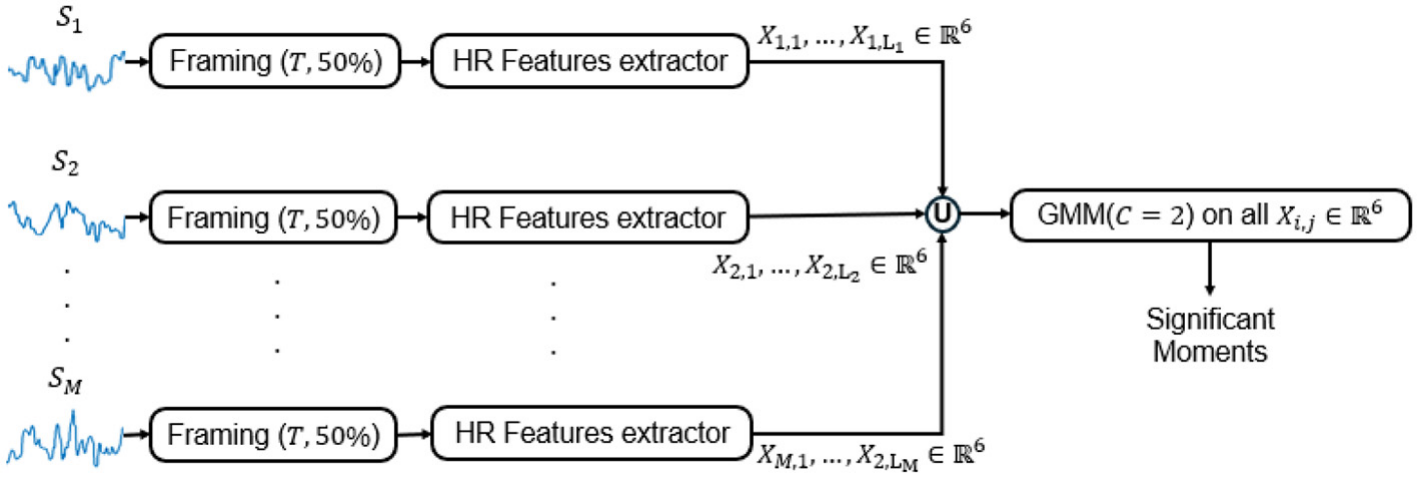
The pipeline designed for tracking the students' emotions based on the HR signal. T is the duration of the chunk.

Feature vectors derived from these metrics were clustered with GMM to detect SMs. For each session, HR feature vectors were clustered to identify outliers, which were interpreted as SMs. The overall process, illustrated in Figure 4, tracks a given student across all teaching sessions *S*_1_, …, *S*_*M*_, with the final set of SMs obtained as the union of all time-window results.

Gaussian Mixture Model (GMM) was chosen because it represents each cluster as a mixture of Gaussian distributions, offering the flexibility to capture clusters that are non-spherical and vary in size within the feature space. In contrast to distance-based methods such as k-means, it yields probabilistic cluster memberships, enabling the identification of borderline cases between “normal” and “specific” moments. This capability to model multimodal feature distributions is well suited to the variability inherent in physiological and behavioral signals observed during classroom activities.

For the EFE analysis, in contrast to HR signal processing, where variable T chunk durations are employed based on flexible guidelines (Kreibig, 2010), we focused on frame-level recognition. This approach was selected due to the demonstrated reliability of using individual facial images for emotion recognition tasks in deep learning models (Li and Deng, 2020). Each processed facial image is passed through a Vision Transformer (ViT) architecture pre-trained for emotion recognition (Dosovitskiy, 2020) on FER2013 (Goodfellow et al., 2013) and subsequently fine-tuned on AffectNet (Mollahosseini et al., 2017). This model was selected based on its reported performance on benchmark datasets, where transformer-based architectures demonstrated superior generalization to unconstrained conditions compared to conventional convolutional networks. Such conditions, including pose variation, partial occlusion, and heterogeneous lighting, are characteristic of natural classroom environments.

The extracted feature vectors are then clustered using a GMM to identify two clusters: one representing typical moments (normal behavior) and the other representing significant deviations (specific moments). The cluster corresponding to SMs is selected based on its outlier characteristics, as it contains frames where the feature vectors deviate substantially from the majority cluster. This allows us to pinpoint moments of emotional or behavioral variation. The extracted feature vectors are then clustered using a GMM to identify the corresponding SM.

The frame-level analysis allows for precise detection of dynamic and subtle emotional variations, ensuring robust identification of significant behavioral patterns while maintaining computational efficiency. This subject-dependent method offers a personalized approach that considers individual differences in emotional state and cultural background. Given that emotional facial expressions can be influenced by a range of factors, this approach enables a more precise and context-aware evaluation of each student's behavioral data.

#### 3.5.3 The processing of data from the questionnaire

The questionnaire yielded two types of data: both the scores obtained on items related to enjoyment, boredom, and anxiety for each session, even though the number of sessions for the three learners varies due to occasional absences. These scores are already a good indicator of the students' emotional state. These scores are supplemented by the students' responses to open-ended questions, which provide an even more precise insight into the emotions experienced during the various classes. Sometimes, the self-reported data do not quite match the scores for enjoyment, boredom, and anxiety. For example, during session 9, Mitch has a high enjoyment score (3.77/5) and a lower boredom score (2.875/5). Based on these two scores, we can say that the dominant emotion for Mitch during this session is enjoyment. However, in his responses to the two open-ended questions, he states: “I was happy to finally start learning passé composé, but that's about it otherwise. I don't feel much normally in class. Just bored. I didn't feel anxious in class today. It was fine, but I was a little bored. I prefer getting called on to stay engaged or my mind wanders and I stop paying attention.” The dominant emotion in his comments is clearly boredom: thus, the self-reported data diverge in this case. In other cases, on the contrary, the self-reported data are completely convergent. The scores and the responses to the two open-ended questions align completely. For instance, during session 6, Oksana has an enjoyment score of 3.88, a boredom score of 2, and an anxiety score of 2.125, and she states: “Today was a good day. I was happy to speak and happy to prepare the text for this class. I wasn't stressed today.” While we sought convergences between the self-reported data, we also aimed to evaluate the convergences between the three components, namely between the HR's SM (Component 1: C1), the EFE's SM (Component 2: C2) and finally with the classroom observations (OBS). These observations are primarily descriptive; they were used to precisely describe the course's progression and to note the students' activities during the different phases of the class.

## 4 Results

### 4.1 Clarifications of experimental results

**Figure 5:**
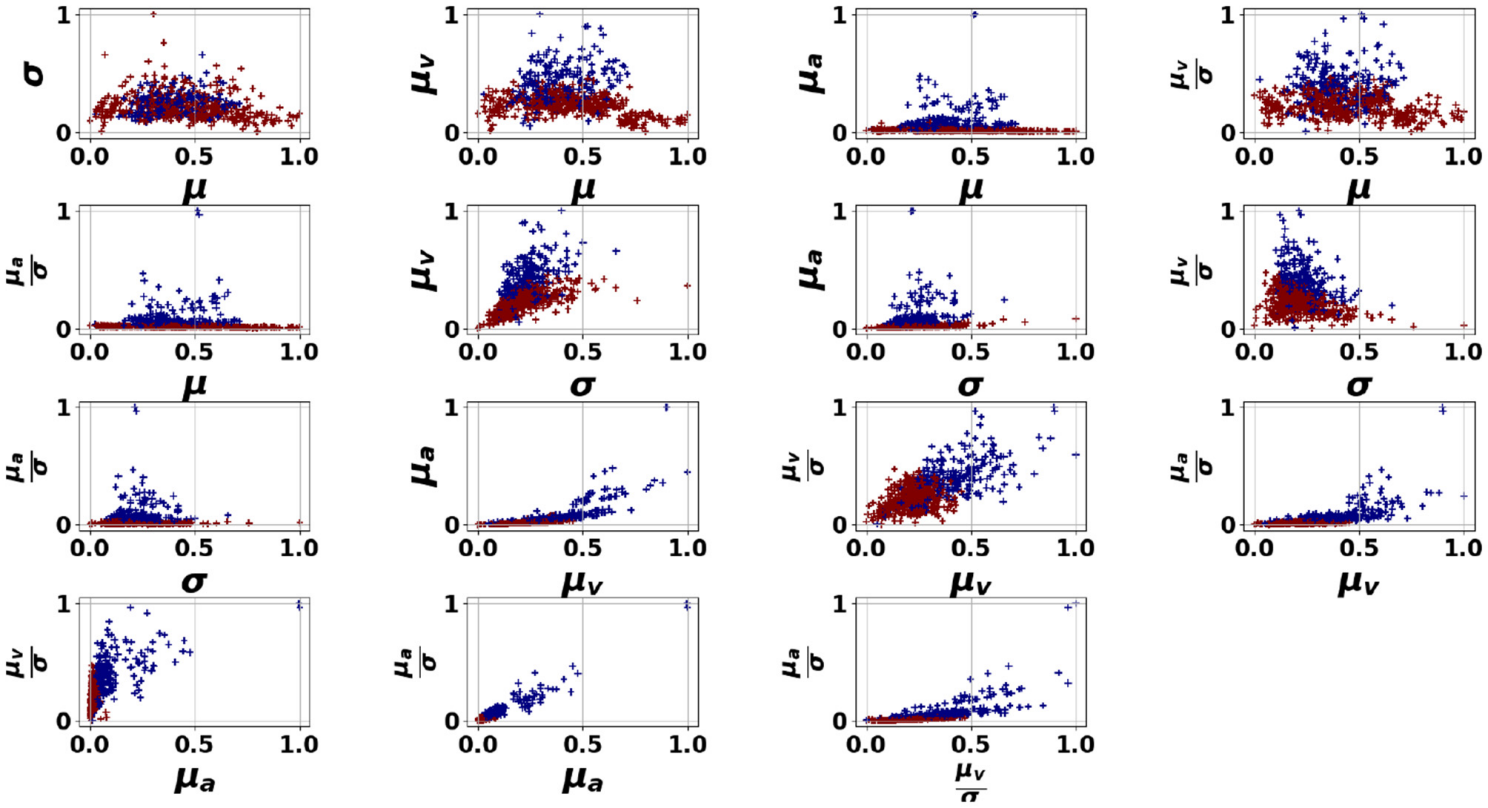
GMM applied on the HR features space for Oksana. μ, σ refer respectively to the mean and the standard deviation of the HR signal, μ_*v*_, μ_*a*_ refer respectively to the mean absolute speed and the mean absolute acceleration of the HR signal.

**Figure 6:**
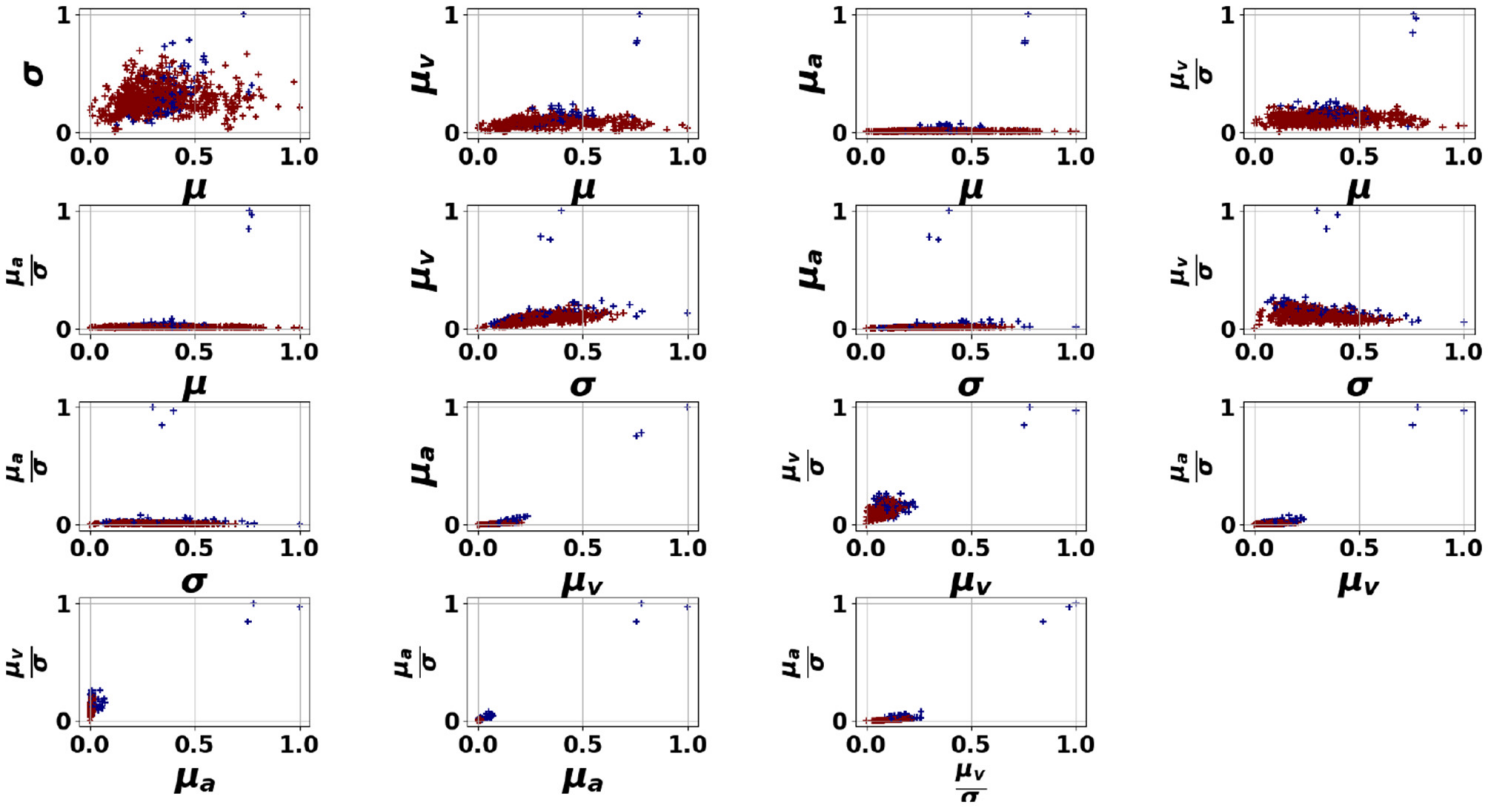
GMM applied on the HR features space for Mitch. μ, σ refer respectively to the mean and the standard deviation of the HR signal, μ_*v*_, μ_*a*_ refer respectively to the mean absolute speed and the mean absolute acceleration of the HR signal.

**Figure 7:**
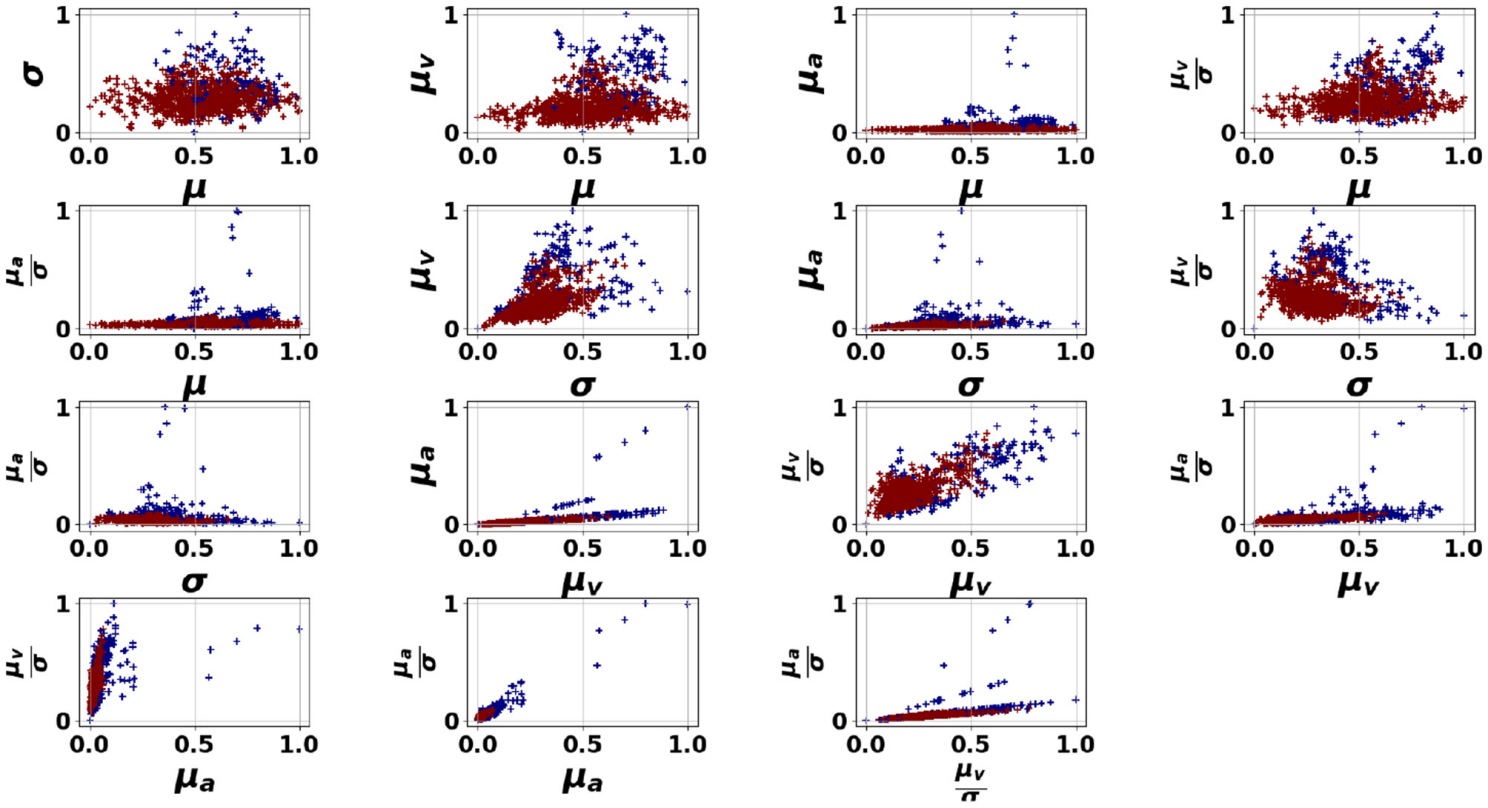
GMM applied on the HR features space for Zeynep. μ, σ refer respectively to the mean and the standard deviation of the HR signal, μ_*v*_, μ_*a*_ refer respectively to the mean absolute speed and the mean absolute acceleration of the HR signal.

We first discuss the experimental results of the proposed pipelines presented in Figure 4, applied to the dataset, in relation to the performance of the HR features in detecting anomalous moments. Figures 5–7 illustrate the HR feature space following an unsupervised clustering via GMM applied to HR features extracted from 150-second segments. This interval was selected as it offered the best balance between temporal resolution and stability of HR features, as discussed in Section 3.4.1. In these figures, we can clearly observe the formation of two clusters for students 1 (Oksana) and 3 (Zeynep). However, student 2 (Mitch) presents a different pattern with a small number of outliers appearing far from the dense area (norm).

**Figure 8:**
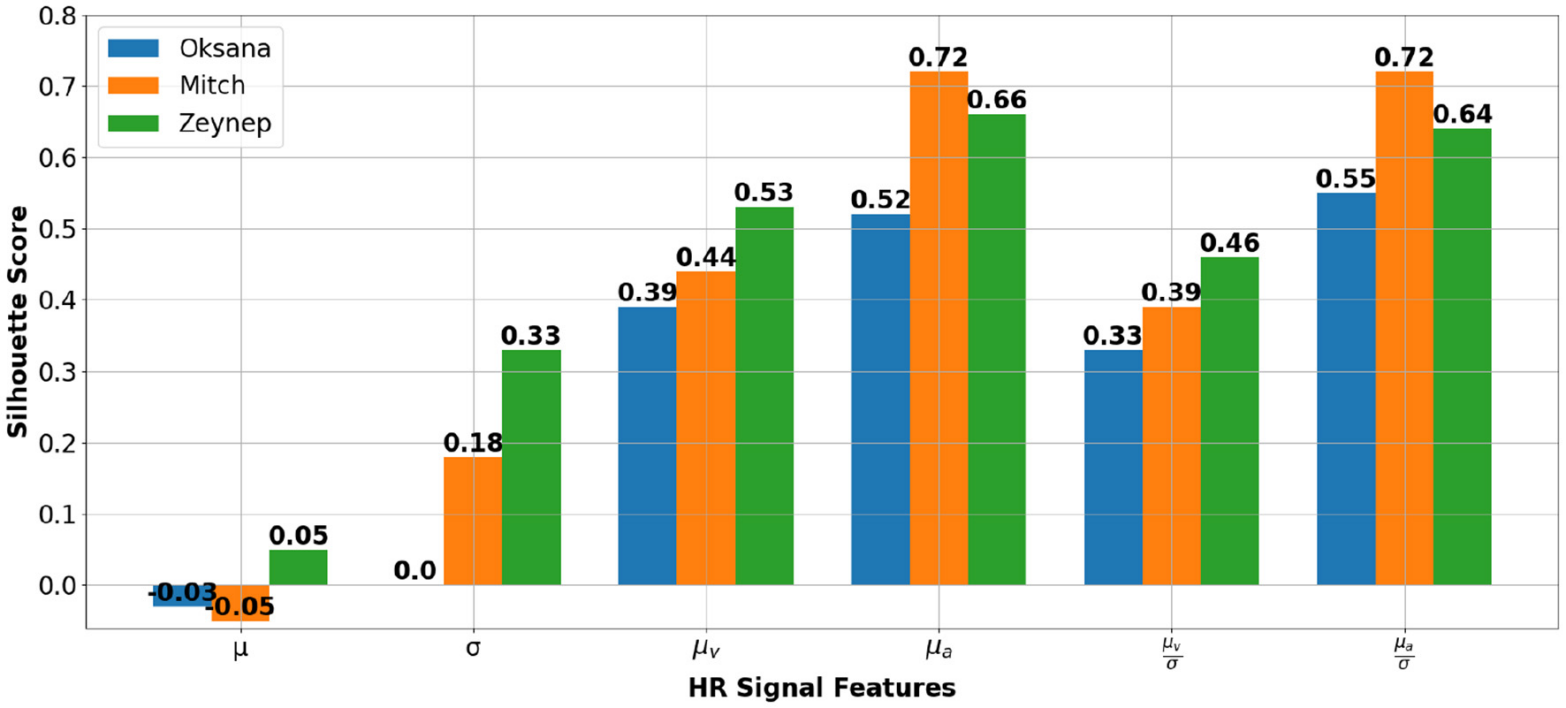
Silhouette scores for each student when using GMM clustering on HR chunks of 2 min 30 seconds.

To evaluate the cohesion and separation of the clusters resulting from the unsupervised GMM, we use the mean of silhouette score metric over all samples (Rousseeuw, 1987). Specifically, for a sample *i* from the data, the silhouette score *s*(*i*) is calculated as follows:


(1) $$\begin{array}{l} s\left(i\right)=\frac{b\left(i\right)-a\left(i\right)}{\max\left(a\left(i\right),b\left(i\right)\right)} \end{array}$$

where *a*(*i*) represents the average distance between *i* and all other points within the same cluster, capturing the intra-cluster cohesion, and *b*(*i*) denotes the minimum average distance between *i* and all points in any other cluster, representing the closest inter-cluster separation. The silhouette score *s*(*i*) ranges between –1 and 1, with values close to 1 indicating well-clustered samples, values near 0 suggesting boundary points between clusters, and values below 0 indicating possible misclassification of *i* to its assigned cluster. In Equation (1), i denotes a single sample data point from the HR feature space illustrated in Figures 5–7. The silhouette score s(i) is calculated for each of these points, and the overall silhouette score shown in Figure 8 corresponds to the average value mean(s(i)) computed across all such data points. Figure 8 presents the silhouette scores for each GMM clustering applied to the three students. Notably, it illustrates the dominance of HR acceleration μ_*a*_ and its normalized value $\frac{\mu_{a}}{\sigma }$ in the clustering process, followed by μ_*v*_ and $\frac{\mu_{v}}{\sigma }$. suggesting that these four features play a significant role in differentiating between the SM of the students and their normal behavior. By focusing on these specific physiological parameters, it might be possible to improve the accuracy of clustering or classification models that rely on HR signals to infer the SMs of the student. This observation is further supported by findings in emotion classification using physiological signals (Mera and Ichimura, 2004), which demonstrated the superior contribution of these four features over the mean and the standard deviation of HR signal.

### 4.2 Convergences between the cross-referenced results of components

**Figure 9:**
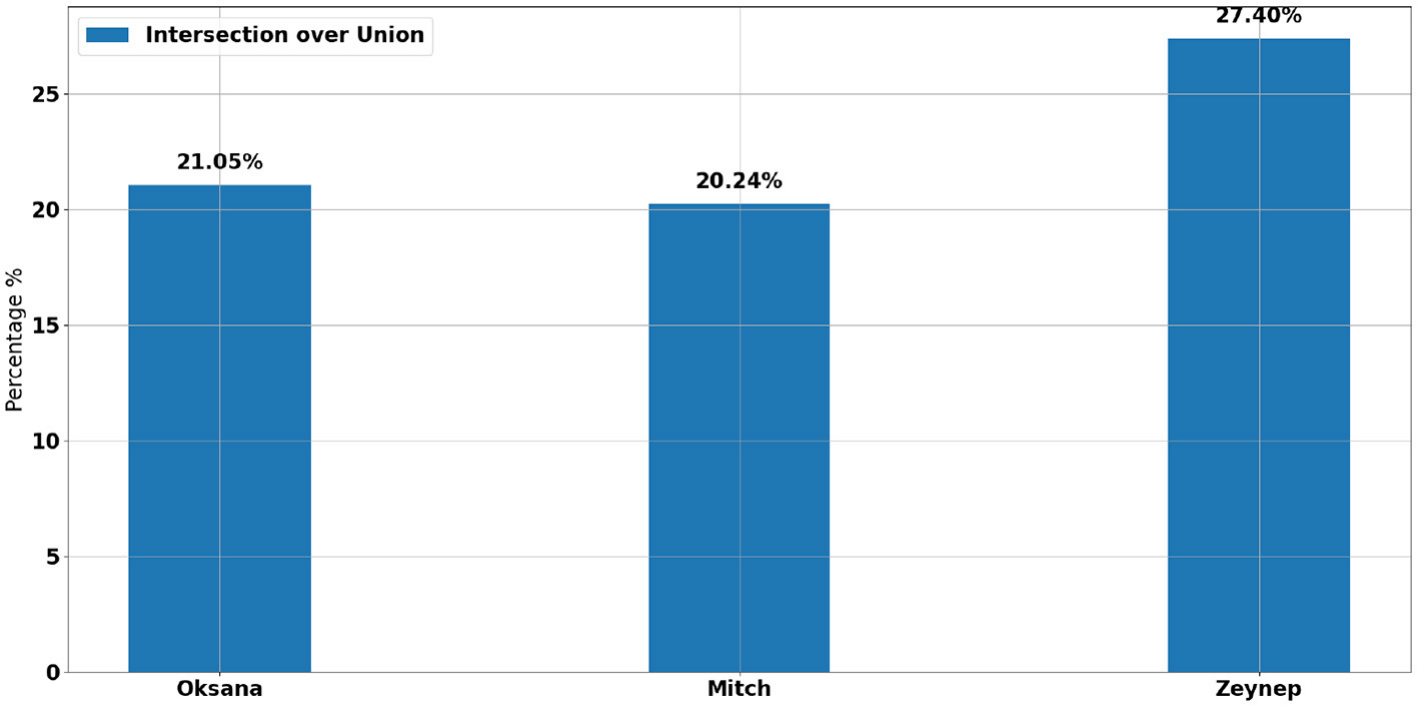
Intersection over Union of HR (Component 1) and EFE (Component 2) SM for each student.

Integrating HR signals (component 1) and EFE (component 2) could improve the detection of students' activity and emotional states. Figure 9 illustrates the percentage of SM detected through the Intersection over Union (IoU) of HR signals and EFE for the three students. This metric quantifies the overlap between the specific moments identified by HR signals and those identified by EFE. The IoU percentages were computed by aligning SMs from both modalities on a common timeline and calculating the ratio of their intersection to their union. This alignment addressed the difference in temporal resolution between modalities, as HR SMs were derived from 150-second windows, whereas EFE SMs were computed at 1 frame per second, ensuring the comparison was not biased by sampling rate differences. It is important to note that only the anomalies in EFE present during the periods of HR signal recording were considered. For Mitch and Oksana, the IoU between the SM of HR signals and EFE is 21.05% and 20.24%, respectively. These moderate overlaps indicate some consistency between the HR data and EFE, although there may be variability in capturing the students' moments of activity and their emotional states. In contrast, Zeynep shows an IoU of 27.40%, the highest among the three students, suggesting a stronger convergence between HR signals and EFE for identifying moments of activity for this student. This comparison highlights that although there is some level of agreement between the two modalities for all students, the extent of this alignment varies, with Zeynep presenting the strongest correlation.

### 4.3 Convergences between the cross-referenced results of components 1 (*C*_1_), 2 (*C*_2_) and the observations of the courses

The results presented in Table 1 illustrate the effectiveness of the HR signal-based method for detecting students' activity and emotional states during teaching sessions. The table provides a comparative analysis of the percentage of convergence between the results by our HR signal-based method (*C*_1_) and the results obtained by our EFE based method (*C*_2_) and finally by the students' observations made during the 16 sessions by an expert in pedagogical methods (OBS).

**Table 1:** The convergences between: *C*_1_ (results obtained by our HR signal-based method) and OBS (observation of the course progression and students' activities); and between *C*_2_ (results obtained by our EFE based method) and OBS; the Multimodal Decision Fusion and OBS.

**Student**	***C*_1_ ∩ *OBS***	***C*_2_ ∩ *OBS***	**Decision fusion ∩ OBS**
Oksana	70%	55%	**80%**
Mitch	72.22%	62.5%	**90%**
Zeynep	54.54%	16.67%	**63.62%**

In addition, fusing both modalities (HR signal-based and EFE-based) through decision fusion achieves the highest convergence rates with expert observations across all students. This outcome highlights that integrating both physiological and behavioral features enhances the accuracy of detecting students' activities and emotional states compared to using unimodal approach.

The questionnaires and open-ended responses, self-report (SR)(*C*_3_) offered valuable insights into the overall emotions experienced by students during the classes, yet they lacked the temporal precision needed to pinpoint specific moments within a session. As a result, it was not possible to statistically cross-reference *C*_3_ with *C*_1_ or *C*_2_, since the latter two modalities enable the identification of specific SMs at precise times during the class. Instead, the *C*_3_ data were used to characterize students' global emotional states across sessions, providing a broader interpretive context for the statistical findings from *C*_1_ and *C*_2_, as discussed later for each student. In contrast, the OBS data—derived from the researcher's detailed classroom notes described in Section 3.1—offered fine-grained temporal information, including exact time stamps and durations, allowing the progression of the lesson to be tracked with high precision.

On the other hand, the OBS are highly detailed, providing precise time and duration indicators to track course progression. These indicators use the same model as *C*_1_ and *C*_2_ with the SMs. Consequently, we were able to cross-reference the data between *C*_1_ and OBS, *C*_2_ and OBS, as well as the multimodal decision fusion of *C*_1_ and *C*_2_ with OBS. The results of these comparisons are presented in Table 1 below.

To answer the *RQ*_1_, for Oksana (Student 1), the agreement between the classroom observations (OBS) and the HR signal-based method (*C*_1_) is significant at 70%, indicating that the HR method is highly effective in objectively detecting when the student is active in class. This convergence shows that the HR method provides a reliable and objective measure of student activity compared to observational methods. In contrast, the convergence between OBS and the EFE-based method (*C*_2_) is lower at 55%, indicating that while the EFE method can reflect classroom activity to some extent, it lacks the precision of the HR-based method. The fusion of these two modalities enhances the accordance to 80%, an average improvement of 17.5% compared to unimodal methods, highlighting the need for multimodal benefits in better detecting SMs at the local time level for this student.

Mitch (Student 2) exhibits a strong agreement of 72.22% between OBS and *C*_1_, reinforcing that the HR method provides a more objective and reliable measure of classroom activity compared to the subjective or observation-based methods. The convergence between *C*_2_ and classroom observations is also relatively strong at 62.5%, but it is still lower than the HR-based measure, highlighting the HR method's superiority in offering a more accurate, objective assessment. The fusion of these two modalities achieves a convergences of 90%, representing an average increase of 22.64% over unimodal methods. This underscores the importance of multimodal approaches in more effectively detecting SMs at the local time level for this student.

For Zeynep (student 3), the HR signal-based method (*C*_1_) shows a moderate agreement with classroom observations at 54.54%, suggesting that while the HR method objectively captures Zeynep's classroom activity, the complexity of her physiological responses may not always align perfectly with direct observations. Nonetheless, the HR method remains more objective than the EFE-based method (*C*_2_), which shows a much lower convergence of 16.67% with classroom observations. While the unimodal performance has decreased for this student, the fusion of these two modalities still enhances convergences by 63.62%, representing an average increase of 28.02% over unimodal methods. This underscores the importance of multimodal approaches in more effectively leveraging the complementary information from the EFE and HR modalities, as well as in detecting SMs at the local time level for this student.

The data in Table 1 highlight the ability of our HR signal-based method to effectively detect 3 students' activity and, at times, emotional states compared to the EFE. However, the fusion of these modalities has led to better detection of SMs at the local time level within the session. The substantial agreement with students' subjective experiences and the precise, direct classroom observations validates the robustness of our approach. This cross-verification reinforces the credibility of our HR signal-based method, demonstrating its applicability and potential to provide objective and insightful data on student activity and emotional states in educational contexts.

### 4.4 Detailed analysis of triangulated results with component 3: focus on each student

**Figure 10:**
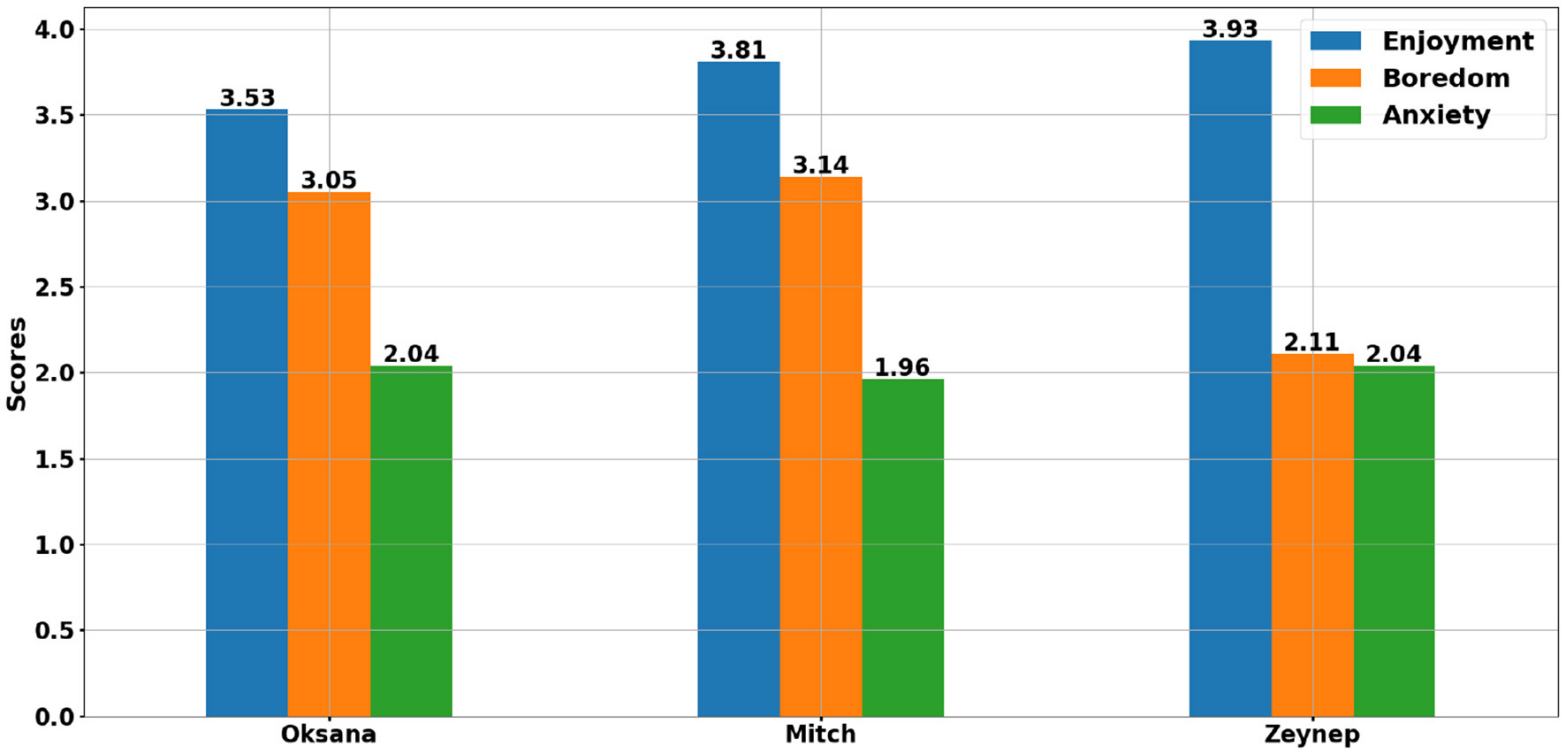
Average scores of the three students for the three emotions across all sessions.

To answer the first part of *RQ*_1_ and *RQ*_2_, we will now focus on the three participants during one particular session. As we explained previously, within component 3, the data were not always convergent. As shown by the Figure 10 of the average scores of the three emotions for the three students, enjoyment is highest. However, the open-ended questions reveal that the dominant emotion for the 14 sessions where Mitch was present is boredom. This observation refers to the most frequent emotion across his individual sessions, whereas the average scores in Figure 10 reflect the mean intensity of each emotion over all sessions; a few sessions with high enjoyment scores can therefore raise the overall average above boredom despite its greater frequency. For Oksana (present at 13 sessions) and Zeynep (present at 14 sessions), enjoyment dominates in the responses to the questions, even though they sometimes experience boredom and anxiety in the French FL class.

We will now examine in more detail one session for each student where the results between the three components and the classroom observations were convergent or semi-convergent. We selected session 14 for Mitch and Oksana where the emotion of enjoyment was predominant for both, and they had moments of shared enjoyment. Session 15 was selected for Zeynep who also reported very high enjoyment.

#### 4.4.1 Emotional contagion between Mitch and Oksana

The results concerning Mitch during session 14 are convergent and semi-convergent across all components and classroom observations. During this session, the dominant emotion for him was enjoyment (which is rare for him): the enjoyment score is higher (3.77) than the boredom score (2.25) and the anxiety score (2). The boredom score during this session is much lower than in the other thirteen sessions, where it hovers around 3 or 3.5. The responses to the two open-ended questions are consistent with these scores, as Mitch wrote: “I felt good throughout the whole class. They were engaging, and their teaching style is better, and I like being able to read and write what I learn.” He did not report any negative emotions.

The convergence between the SMs of Component 1 is almost total because 3 out of the 5 SMs correspond to Mitch's very active participation in the class, as shown by the observation of the class progression. He is very engaged in the different tasks and highly motivated. The SMs correspond to the moment when he stood up to read a very personal text about what the meaning of life is for him; during another SM, he worked with the teacher. For the third SM, Mitch did a group activity with Oksana.

The convergence between the observation of the class proceedings and Component 2 (EFE) is not complete because, out of 18 SMs in the EFE, only 7 correspond to a significant element concerning Mitch during the class. The convergence between the SMs of component 1 and those of component 2 is also semi-convergent, as 4 SMs are shared by both components, and only 2 out of the 4 SMs correspond to a significant element of what Mitch did during the session.

One of the factors that might explain the intense enjoyment Mitch felt during this session is likely related to the fact that the class was led by two student interns from the Master's program in “Language Didactics” and that they did not use the Neurolinguistic Approach (NLA) method which Guillaume, the French FL teacher for the course, relies on. They conducted a class with the theme “Shitty Life.” Mitch expressed the boredom he often felt due to the repetitive structure of NLA with its different phases. However, this boredom is mainly linked to the heterogeneity of the group of learners. Mitch, Oksana, and Zeynep were bored during most of the other sessions because they found the tasks too easy, too simple for them.

Oksana experienced very strong enjoyment during this same session 14. Her enjoyment score is 4, while the boredom (2.25) and anxiety (2) scores are lower. She responded to the open-ended questions by saying, “Today was an interesting class,” and she did not report any negative emotions. Regarding component 1 (HR), the SMs converge with the observations of the class proceedings. Oksana was very active during these SMs and did most of the tasks with Mitch. Regarding component 2 (EFE), only 5 out of the 12 SMs correspond to significant elements of what she did in class. Six SMs overlap between components 1 and 2, indicating that these results are semi-convergent. Among these 6 SMs, only 4 reflect Oksana's active participation in the class. This semi-convergence means that Oksana's active participation in class is not always aligned with the SMs. It is clear that her internal emotional reactions are not always visible through external observations and that she is not always aware of them either. Therefore, there may be SMs that do not match either her actions in class or what she has reported in the responses to open-ended questions.

#### 4.4.2 Zeynep, a strong emotional engagement

The dominant emotion for Zeynep during the 14 sessions she attended in the FL French course was clearly enjoyment. Therefore, we selected session 15, where the results across different components were convergent. Her enjoyment score for session 15 (3.88) is thus higher than those for boredom (2.125) and anxiety (2.125). She responded very positively in French to the open-ended questions in the questionnaire: “I am happy to learn new things. We talked a lot today. Original version in French:” Je suis heureuse pour apprendre des nouveaux choses. On a parlé beaucoup aujourd “hui.” She did not report any negative emotions experienced.

Out of the 17 SMs from component 1 (HR), three correspond to significant elements noted in the observations of the course's progression concerning Zeynep. Indeed, at these moments, she was highly engaged in oral interactions during the oral phase of the NLA. She was modeling a sentence related to the most important person to her when she was a child. She spoke about her grandfather, with whom she grew up. This was, therefore, a very emotionally intense moment for her. During the other two SMs, she was interacting with other learners, particularly with Oksana. The SMs from components 1 and 2 almost entirely converge, as 16 SMs from component 1 out of 17 are found in six SMs from component 2 (EFE). However, only 4 SMs among these 16 correspond to a significant activity by Zeynep during the class. This is why we can say that the results between these two components are semi-convergent.

We speculate that the strong enjoyment Zeynep experienced is likely due to the emotional contagion that emerged during her interactions with Oksana in the oral phase of the NLA, as well as the fact that she was talking about emotionally significant topics for her, which boosted her motivation and engagement in oral tasks.

## 5 Discussion

This study aimed to identify the emotions that students experience during a French FL course over the progression of an entire semester in a university setting using a multimodal approach. Addressing *RQ*_1_, which concerns the relationship between scores obtained from classroom observations and measures of physiological reactions (HR variation) and EFE, results varied significantly for each of the three students, confirming the findings in previous researchs (Bielak and Mystkowska-Wiertelak, 2024; Elahi Shirvan and Talebzadeh, 2018; Elahi Shirvan et al., 2020; Gregersen et al., 2014).

It is *RQ*_2_ that helps refine the initial results obtained from *RQ*_1_. Indeed, the convergence between all data sources was generally strong. It seems that objective data sometimes capture emotional states of which learners are not always aware, and thus may not verbalize in self-perception reports. The classes lasted twice 1 hour and 30 minutes, which are long periods during which emotions fluctuate greatly. Students interviewed after the class may not remember everything that happened during those 3 h. This is why the measurement of their EFE and HR variation, as well as classroom observations, were triangulated with their FLE and FLCB scores and their responses to open-ended questions to gain a more holistic and precise view of the emotions experienced over such a long period.

The moments of convergence between all data and the three components of emotion are primarily related, to specific moments in the class that the student remembers and that are often characterized by strong enjoyment linked to stimulating and collaborative group activities. These findings align with those of Nozawa et al. (2021), which demonstrated positive emotional dynamics linked to pair-work.

It also seems that boredom, which is reflected in a decrease in student activity during class, corresponds to the presence of fewer SMs identified in HR variation and EFE. This supports the findings of Li et al. (2022), which showed the very negative effects of boredom on language learners' motivation and their WTC.

Anxiety was detected in the AMs during oral activities performed in front of other group members during the oral phase. This confirms the finding in Gregersen et al. (2014) and Dewaele and MacIntyre (2014) that oral presentations generate mild to high anxiety in the FL classroom.

The three students in this study found the activities related to the NLA method boring and too repetitive because their level in French FL was much more advanced than that of the other students in the group. Agrawal et al. (2022) and Li (2021) explained that boredom occurs with repetitive, under-challenging tasks when learners feel that they do not learn anything new. However, we observed that during oral activities and when discussing emotionally intense topics (as in Unit 3 of the semester, which involved talking about events that marked our lives through anecdotes and unforgettable moments that shaped our past), the students were very engaged, and the results across the three components of the study converged during these moments.

The multimodal datastream allowed us to capture moments of convergence between the specific moments detected by physiological measurements and classroom observations, with learners' self-reports through questionnaires helping us interpret these results. These reflected episodes of positive emotional contagion between Oksana and Mitch during oral task activities and between Oksana and Zeynep (Moskowitz and Dewaele, 2021; Talebzadeh et al., 2020). They were highly engaged together in the different tasks proposed by the teachers. This confirms the findings in Dewaele and MacIntyre (2014) and Bielak and Mystkowska-Wiertelak (2024) that working with a partner can be a powerful source of enjoyment and can create a sense of solidarity and empathy. The ability to communicate with peers and overcoming the fear of making mistakes in a positive environment is vital (Elahi Shirvan et al., 2020). This positive emotional contagion experienced within a small group can lead to significant engagement in the task and even to a state of flow (Nozawa et al., 2021). We could argue that where Nozawa et al. (2021) caught evidence of brain synchronization between partners, we found evidence of “heart synchronization.”

Positive emotions can help sustain motivation (MacIntyre et al., 2019). More specifically, FLE and levels of motivation flourish together (Dewaele and Proietti Ergün, 2020; Wang et al., 2023), while FLCA has the opposite relationship (Dewaele and Proietti Ergün, 2020).

What matters to learners is that the activity allows them to collaborate with students they enjoy working with and enables them to express genuine emotions and feelings (Bielak and Mystkowska-Wiertelak, 2024). However, the activity must be sufficiently stimulating and challenging for them to engage with it. Any activity deemed too easy and repetitive risks being considered uninteresting, leading to a drop in engagement.

The multimodal method developed in the present study for tracking student emotions is both simple and easy to deploy, effectively addressing the complexities of classroom environments. By using low-cost sensors to capture HR signals and EFE, we aimed to create a dataset and to explore a pipeline capable of identifying SMs based on unsupervised clustering at the student level. n other words, our approach prioritizes analyzing each student's physiological and behavioral variations in relation to their baseline emotional state, rather than generalizing across all students to derive SMs for each teaching session. This individualized method is both accessible and reproducible, ensuring tailored and reliable insights. For the analysis of HR signals, based on literature (Kreibig, 2010; Wu, 2023), we selected time windows ranging from 60 to 210 seconds with a 50% overlap to capture the temporal variations in physiological responses. We employed unsupervised GMM clustering for each student individually, taking into account cultural and gender differences to reveal distinct emotions.

This approach not only improved the detection of SM but also enhanced the overall robustness of our emotions tracking system. The silhouette scores used to evaluate the cohesion and separation of clusters confirmed the effectiveness of our clustering strategy, particularly highlighting the importance of HR acceleration and speed parameters in distinguishing levels of engagement.

In contrast to the temporal processing of HR signals, the analysis of EFE focused on image-level recognition. Using a pre-trained ViT for emotion recognition, we clustered the resulting feature vectors with GMM to identify specific moments. This demonstrated the feasibility of using advanced neural network architectures for real-time emotions tracking in educational settings.

Overall, the proposed multimodal pipeline, combining HR signals and EFE, and cross-referenced with questionnaires and classroom observations, would provide a comprehensive framework for understanding students' emotions. The experimental results highlight the potential of multimodal pattern recognition to enhance our understanding of the emotions experienced by students during FL learning. Further research may consider lip emotion recognition models which can be employed as a substitute for EFE and have the advantage of maintaining participants' anonymity.

However, this study is not without its limitations. Firstly, longitudinal research in a naturalistic setting involving a large amount of multimodal data cannot be carried out on large groups and all longitudinal research inevitably suffers from attrition over time (Dörnyei, 2007). Moreover, the use of multiple tools increases the risk of technological malfunction which can lead to further loss of participants in the experiment (Hoemann et al., 2023). However, small sample sizes are the norm in research inspired by Complex Dynamic Systems Theory (Hiver et al., 2022) where the aim is to collect rich and detailed longitudinal data about unique individuals.

Secondly, utilizing HR signals allows us to track individual emotions continuously throughout the session, even when visual cues might occasionally be obscured due to students' head poses or obstructions by the teacher. Additionally, these two modalities provide distinctly different types of information: EFE reflect behavioral responses to emotional states, while HR signals represent physiological reactions that offer more objective measures for emotion recognition systems (Wang et al., 2022; Wu, 2023). However, EFE may be unreliable as individuals can consciously control these physical manifestations to hide their true emotions, a phenomenon known as social masking (Shu et al., 2018; Wu, 2023). Therefore, while HR monitoring is more intrusive, it is also more effective in accurately tracking student emotions. The integration of these two modalities has proven to be more reliable in determining emotional states.

The study also involved a small number of students because implementing such a protocol in the classroom presents various technical challenges and can be potentially disruptive. We should point out that, due to the lack of data, we opted for unsupervised clustering to identify SM based on the two modalities stated previously. In a scenario where data is abundant and live tracking of student emotions is preferred, we would suggest isolating an initial session to establish a baseline for normal and SM for each student, followed by using a sliding time window in subsequent sessions for real-time emotion tracking.

In a future study, it would also be interesting to show the videos to the students and discuss with them some time after the class as is the case in idiodynamic studies (MacIntyre and Ducker, 2022), showing them the SM detected both from the HR signal and by the camera for the EFE.

## 6 Conclusion

The originality of the present study lies in its interdisciplinary approach and in the development of new tools to capture fleeting emotions. To the best of our knowledge, no previous study has integrated emotional facial expressions (EFE), heart rate (HR) signals, classroom observations, and self-reports—over an extensive duration and across multiple sessions, while considering the complexities inherent in real-world teaching environments. The novelty of this multimodal approach, combined with the absence of comparable studies using all modalities in similar contexts, precludes direct comparisons with state-of-the-art methods. Despite this limitation, our study provide valuable insights into student's emotions and offer new perspectives for future research in this area.

The decision to combine applied linguistics, language and culture didactics, artificial intelligence studies, computer engineering, automation, and signal processing allowed us to expand the range of dependent variables and to shed light on the complex dynamic system of language learners' emotions at work in their classrooms. The rich stream of multimodal data collected from authentic interactions between three learners in one intact classroom over 16 sessions increased the ecological validity of the analyses. The large quantity of data also allowed us to zoom in on episodes of particular interest, namely peaks and drops in the various emotions and especially moments of convergence between heart rates, facial expressions and self-reported data. These moments could be interpreted in light of the tasks being performed and the empathy with the partner.

This study provides researchers with new tools to capture the many manifestations of dynamic FL learner emotions and represents a move away from exclusive reliance on learners' self-reports. The well-known phenomenon of emotional contagion could thus be observed in real-time across modalities and its sources could be identified.

## Data Availability

The datasets generated during the current study are available from the corresponding author upon reasonable request, in compliance with GDPR requirements.
