# Supplementary material for: Heart rates, facial expressions and self-reports: a multimodal longitudinal approach of learners' emotions in the foreign language classroom
Source: Front Artif Intell. 2025 Oct 1;8:1604110. doi: 10.3389/frai.2025.1604110 (PMC12521460; doi:10.3389/frai.2025.1604110)
Supplement: Supplementary file 1 [file Data_Sheet_1.pdf]

Nom : ……………………………………………………………………………

Prénom : …………………………………………………………………………..

Attention, toutes les informations que vous allez indiquer ici seront confidentielles, et elles seront anonymées ensuite.

Réponds aux questions sur ta classe de français cette année en cochant la bonne réponse.

**1. La/le prof de français est encourageant/e.**<br><br>**1. The French teacher is encouraging.**	**❑ Pas du tout d'accord/ I strongly disagree**	**❑ Pas d'accord/ I disagree**	**❑ Je ne suis ni d'accord ni Pas d'accord/ I have no opinion about this**	**❑ D'accord/ I agree**	**❑ Tout à fait d'accord / I strongly agree**
**2. La/le prof de français est gentil/le.**<br><br>**2. The French teacher is friendly.**	**❑ Pas du tout d'accord/ I strongly disagree**	**❑ Pas d'accord/ I disagree**	**❑ Je ne suis ni d'accord ni Pas d'accord/ I have no opinion about this**	**❑ D'accord/ I agree**	**❑ Tout à fait d'accord / I strongly agree**
**3. La/le prof de français m'aide beaucoup.**<br><br>**3. The French teacher is supportive.**	**❑ Pas du tout d'accord/ I strongly disagree**	**❑ Pas d'accord/ I disagree**	**❑ Je ne suis ni d'accord ni Pas d'accord/ I have no opinion about this**	**❑ D'accord/ I agree**	**❑ Tout à fait d'accord / I strongly agree**
**4. J'aime ce cours de français.**<br><br>**4. I enjoy this French class.**	**❑ Pas du tout d'accord/ I strongly disagree**	**❑ Pas d'accord/ I disagree**	**❑ Je ne suis ni d'accord ni Pas d'accord/ I have no opinion about this**	**❑ D'accord/ I agree**	**❑ Tout à fait d'accord / I strongly agree**

**5. J'ai appris des choses intéressantes en classe de français.**<br><br>**5. I've learned interesting things in French class.**	❑ **Pas du tout d'accord/ I strongly disagree**	❑ **Pas d'accord/ I disagree**	❑ **Je ne suis ni d'accord ni Pas d'accord/ I have no opinion about this**	❑ **D'accord/ I agree**	❑ **Tout à fait d'accord / I strongly agree**
**6. Je suis content.e de mes progrès en français.**<br><br>**6. I am proud of my accomplishments in French.**	❑ **Pas du tout d'accord/ I strongly disagree**	❑ **Pas d'accord/ I disagree**	❑ **Je ne suis ni d'accord ni Pas d'accord/ I have no opinion about this**	❑ **D'accord/ I agree**	❑ **Tout à fait d'accord / I strongly agree**
**7. Nous sommes comme une équipe en cours de français.**<br><br>**7. We form a tight group in the French class.**	❑ **Pas du tout d'accord/ I strongly disagree**	❑ **Pas d'accord/ I disagree**	❑ **Je ne suis ni d'accord ni Pas d'accord/ I have no opinion about this**	❑ **D'accord/ I agree**	❑ **Tout à fait d'accord / I strongly agree**
**8. Nous rions beaucoup en classe de français.**<br><br>**8. We laugh a lot during French class.**	❑ **Pas du tout d'accord/ I strongly disagree**	❑ **Pas d'accord/ I disagree**	❑ **Je ne suis ni d'accord ni Pas d'accord/ I have no opinion about this**	❑ **D'accord/ I agree**	❑ **Tout à fait d'accord / I strongly agree**
**9. Nous avons nos propres blagues à nous en cours de français.**<br><br>**9. We have common 'legends,' such as running**<br>**jokes in the French class.**	❑ **Pas du tout d'accord/ I strongly disagree**	❑ **Pas d'accord/ I disagree**	❑ **Je ne suis ni d'accord ni Pas d'accord/ I have no opinion about this**	❑ **D'accord/ I agree**	❑ **Tout à fait d'accord / I strongly agree**
**10. Le cours de français m'ennuie.**<br><br>**10. The french class bores me.**	❑ **Pas du tout d'accord/ I strongly disagree**	❑ **Pas d'accord/ I disagree**	❑ **Je ne suis ni d'accord ni Pas d'accord/ I have no opinion about this**	❑ **D'accord/ I agree**	❑ **Tout à fait d'accord / I strongly agree**

**11. Je commence à bailler dans le cours de français parce que je m'ennuie.**<br><br>**11. I start yawning in french class because I'm so bored.**	❑ **Pas du tout d'accord/ I strongly disagree**	❑ **Pas d'accord/ I disagree**	❑ **Je ne suis ni d'accord ni Pas d'accord/ I have no opinion about this**	❑ **D'accord/ I agree**	❑ **Tout à fait d'accord / I strongly agree**
**12. Je suis dans la lune dans le cours de français.**<br><br>**12. My mind begins to wander in the french class.**	❑ **Pas du tout d'accord/ I strongly disagree**	❑ **Pas d'accord/ I disagree**	❑ **Je ne suis ni d'accord ni Pas d'accord/ I have no opinion about this**	❑ **D'accord/ I agree**	❑ **Tout à fait d'accord / I strongly agree**
**13. Je ne suis que physiquement présent dans le cours de français, mon esprit est à l'extérieur du cours de français.**<br><br>**13. I am only physically in the classroom, while my mind is wandering outside the french class.**	❑ **Pas du tout d'accord/ I strongly disagree**	❑ **Pas d'accord/ I disagree**	❑ **Je ne suis ni d'accord ni Pas d'accord/ I have no opinion about this**	❑ **D'accord/ I agree**	❑ **Tout à fait d'accord / I strongly agree**
**14. J'ai du mal à me concentrer dans le cours de français.**<br><br>**14. It is difficult for me to concentrate in the french class.**	❑ **Pas du tout d'accord/ I strongly disagree**	❑ **Pas d'accord/ I disagree**	❑ **Je ne suis ni d'accord ni Pas d'accord/ I have no opinion about this**	❑ **D'accord/ I agree**	❑ **Tout à fait d'accord / I strongly agree**
**15. Le temps passe lentement dans le cours de français.**<br><br>**15. Time is dragging on in french class.**	❑ **Pas du tout d'accord/ I strongly disagree**	❑ **Pas d'accord/ I disagree**	❑ **Je ne suis ni d'accord ni Pas d'accord/ I have no opinion about this**	❑ **D'accord/ I agree**	❑ **Tout à fait d'accord / I strongly agree**
**16. Je m'agite et je ne peux pas attendre la fin du cours de français.**	❑ **Pas du tout d'accord/ I**	❑ **Pas d'accord/ I disagree**	❑ **Je ne suis ni d'accord ni Pas d'accord/**	❑ **D'accord/ I agree**	❑ **Tout à fait d'accord / I**

16. I get restless and can't wait for the french class to end.	strongly disagree		I have no opinion about this		strongly agree
17. Je pense toujours que le temps du cours serait plus utile pour faire autre chose.<br><br>17. I always think about what else I might be doing to kill the time rather than sitting in this French class.	❑ Pas du tout d'accord/ I strongly disagree	❑ Pas d'accord/ I disagree	❑ Je ne suis ni d'accord ni Pas d'accord/ I have no opinion about this	❑ D'accord/ I agree	❑ Tout à fait d'accord / I strongly agree
18. Même si je suis bien préparé.e pour le cours de français, je me sens anxieux.<br><br>18. Even if I am well prepared for French class, I feel anxious about it.	❑ Pas du tout d'accord/ I strongly disagree	❑ Pas d'accord/ I disagree	❑ Je ne suis ni d'accord ni Pas d'accord/ I have no opinion about this	❑ D'accord/ I agree	❑ Tout à fait d'accord / I strongly agree
19. J'ai toujours l'impression que les autres élèves parlent mieux français que moi.<br><br>19. I always feel that the other students speak the French better than I do.	❑ Pas du tout d'accord/ I strongly disagree	❑ Pas d'accord/ I disagree	❑ Je ne suis ni d'accord ni Pas d'accord/ I have no opinion about this	❑ D'accord/ I agree	❑ Tout à fait d'accord / I strongly agree
20. Je peux sentir mon cœur battre quand je vais être appelé.e en cours de français.<br><br>20. I can feel my heart pounding when I'm going to be called on in French class.	❑ Pas du tout d'accord/ I strongly disagree	❑ Pas d'accord/ I disagree	❑ Je ne suis ni d'accord ni Pas d'accord/ I have no opinion about this	❑ D'accord/ I agree	❑ Tout à fait d'accord / I strongly agree
21. Je n'ai pas peur de faire des erreurs en cours de français.	❑ Pas du tout d'accord/ I strongly disagree	❑ Pas d'accord/ I disagree	❑ Je ne suis ni d'accord ni Pas d'accord/ I have no	❑ D'accord/ I agree	❑ Tout à fait d'accord / I strongly agree

**21. I don't worry about making mistakes in French class.**			**opinion about this**		
**22. Je me sens confiant.e lorsque je parle en cours de français.**<br><br>**22. I feel confident when I speak in French class.**	**❑ Pas du tout d'accord/ I strongly disagree**	**❑ Pas d'accord/ I disagree**	**❑ Je ne suis ni d'accord ni Pas d'accord/ I have no opinion about this**	**❑ D'accord/ I agree**	**❑ Tout à fait d'accord / I strongly agree**
**23. Je deviens nerveux/se et tout se mélange dans ma tête lorsque je parle dans mon cours de français.**<br><br>**23. I get nervous and confused when I am speaking in my French class.**	**❑ Pas du tout d'accord/ I strongly disagree**	**❑ Pas d'accord/ I disagree**	**❑ Je ne suis ni d'accord ni Pas d'accord/ I have no opinion about this**	**❑ D'accord/ I agree**	**❑ Tout à fait d'accord / I strongly agree**
**24. Je commence à paniquer quand je dois parler sans préparation en cours de français.**<br><br>**24. I start to panic when I have to speak without preparation in French class.**	**❑ Pas du tout d'accord/ I strongly disagree**	**❑ Pas d'accord/ I disagree**	**❑ Je ne suis ni d'accord ni Pas d'accord/ I have no opinion about this**	**❑ D'accord/ I agree**	**❑ Tout à fait d'accord / I strongly agree**
**25. Je ne suis pas à l'aise de proposer des réponses dans mon cours de français.**<br><br>**25. It embarrasses me to volunteer answers in my French class.**	**❑ Pas du tout d'accord/ I strongly disagree**	**❑ Pas d'accord/ I disagree**	**❑ Je ne suis ni d'accord ni Pas d'accord/ I have no opinion about this**	**❑ D'accord/ I agree**	**❑ Tout à fait d'accord / I strongly agree**

Réponds à ces deux questions librement (ce que tu as écrit ici sera rendu anonyme et personne à l'intérieur de l'université ne lira tes réponses, c'est uniquement pour les chercheuses qui font ce travail)

- Décris une situation en classe **de français** où tu t'es vraiment senti bien et explique ce que tu as ressenti à ce moment-là avec le plus de détails possibles/ Describe a

moment when you felt really good in **French** class and what did you feel at that moment with the most details.

………………………………………………………………………………………………………
………………………………………………………………………………………………………
………………………………………………………………………………………………………
………………………………………………………………………………………………………
………………………………………………………………………………………………………
………………………………………………………………………………………………………
………………………………………………………………………………………………………
………………………………………………………………………………………………………
………………………………………………………………………………………………………
………………………………………………………………………………………………………

- Décris une situation en classe **de français** où tu t'es vraiment senti anxieux ou anxieuse (stressé, stressée) et explique ce que tu as ressenti à ce moment-là avec le plus de détails possibles. / Describe a moment when you felt really anxious (stressed) in **French** class and what did you feel at that moment with the most details.

………………………………………………………………………………………………………
………………………………………………………………………………………………………
………………………………………………………………………………………………………
………………………………………………………………………………………………………
………………………………………………………………………………………………………
………………………………………………………………………………………………………
………………………………………………………………………………………………………
………………………………………………………………………………………………………
………………………………………………………………………………………………………
………………………………………………………………………………………………………
